# Delineation of the Urban-Rural Boundary through Data Fusion: Applications to Improve Urban and Rural Environments and Promote Intensive and Healthy Urban Development

**DOI:** 10.3390/ijerph18137180

**Published:** 2021-07-05

**Authors:** Jun Zhang, Xiaodie Yuan, Xueping Tan, Xue Zhang

**Affiliations:** School of Architecture and Planning, Yunnan University, Kunming 650500, China; xdy@mail.ynu.edu.cn (X.Y.); xuepingtan@163.com (X.T.); zhangxue86097@163.com (X.Z.)

**Keywords:** urban health, POI, urban environment, urban and rural development, urban and rural fringe

## Abstract

As one of the most important methods for limiting urban sprawl, the accurate delineation of the urban–rural boundary not only promotes the intensive use of urban resources, but also helps to alleviate the urban issues caused by urban sprawl, realizing the intensive and healthy development of urban cities. Previous studies on delineating urban–rural boundaries were only based on the level of urban and rural development reflected by night-time light (NTL) data, ignoring the differences in the spatial development between urban and rural areas; so, the comprehensive consideration of NTL and point of interest (POI) data can help improve the accuracy of urban–rural boundary delineation. In this study, the NTL and POI data were fused using wavelet transform, and then the urban–rural boundary before and after data fusion was delineated by multiresolution segmentation. Finally, the delineation results were verified. The verification result shows that the accuracy of delineating the urban–rural boundary using only NTL data is 84.20%, and the Kappa value is 0.6549; the accuracy using the fusion of NTL and POI data on the basis of wavelet transform is 93.2%, and the Kappa value is 0.8132. Therefore, we concluded that the proposed method of using wavelet transform to fuse NTL and POI data considers the differences between urban and rural development, which significantly improves the accuracy of the delineation of urban–rural boundaries. Accurate delineation of urban–rural boundaries is helpful for optimizing internal spatial structure in both urban and rural areas, alleviating environmental problems resulting from urban development, assisting the formulation of development policies for urban and rural fringes, and promoting the intensive and healthy development of urban areas.

## 1. Introduction

As a boundary that separates urban from rural areas, the urban–rural boundary refers to a city or region that is constantly changing in the process of urban expansion [1]. Therefore, delineating the urban–rural boundary has become one of the most important connotations of new urbanization, and is important for alleviating various urban issues [2]. However, rapid urbanization construction is mainly driven by capital, which inevitably leads to the disorderly expansion of cities. The direct manifestation of a disorderly expansion is those cities constantly consumes rural land resources, and the use rate of these resources is very low [3,4]. Therefore, the accurate delineation of the urban–rural boundary can be used to effectively curb the rural resource plundering by urban areas, and improve the relationship between urban and rural areas, so as to promote the intensive use of urban resources and achieve the healthy development of cities [5].

Although rapid urbanization contributes to the rapid development of a regional economy and the rapid expansion of urban areas to rural areas, it has also created urban–rural environmental problems, including low efficiency of land use, environmental pollution, ecosystem damage, housing tension, traffic congestion, and other urban problems which are increasingly prominent and may seriously affect the use of urban resources and the high-quality development of urban cities. These problems all result from the disorderly expansion of a city caused by a chaotic urban–rural boundary [6,7]. As a result of the imbalance between rapid urban development and urban governance, these urban environmental problems have gradually evolved into serious urban issues that plague urban planners [8]. One method of solving these urban issues is to intensively use urban resources and restrict the disorderly expansion of cities to alleviate the pressure caused by rapid urban development, and then realize the healthy development of urban cities [9]. However, at present, urban development resources are wasted, mainly manifested in the disorderly expansion of urban to rural areas [10]. For example, under the background of urban integration, many new cities have been built, but the use rate of these new cities is extremely low, resulting in the emergence of many “ghost towns” [11]. In summary, unreasonable urban expansion directly leads to the excessive waste of urban resources and affects the healthy development of urban and rural areas. Therefore, the accurate delineation of the urban–rural boundary is conducive to curbing disorderly urban expansion and promoting the intensive and efficient use of resources to achieve the healthy development of urban cities [12]. 

With the continuous progress of the national territory development plan, the goal of optimizing urban space, breaking through the dual urban–rural structure, and realizing the rapid and healthy development of cities was introduced [13,14]. Therefore, the accurate delineation of urban–rural boundaries helps with distinguishing urban from rural areas for the formulation of reasonable urban and rural development planning methods, to then further alleviate the environmental problems in the process of urban and rural development, and achieve the healthy development of urban and rural areas [15]. However, due to the significant differences between urban and rural areas in terms of the economic development level and infrastructure, urban–rural boundaries were previously delineated based on economic, population, and other statistical data [16]. In previous studies, the statistical data of the whole urban and rural areas were generally divided into different levels, with the higher level being the urban area and the lower level being the rural area, then the urban and rural areas were delineated. However, such delineation of urban–rural boundaries is highly subjective and less accuracy, which is why the delineation of the urban–rural boundary based on statistical data has been gradually replaced [17].

As data that can reflect the differences in urban spatial surface information, urban remote sensing has gradually replaced the use of traditional statistical data, such as administrative indicators, economic, and population statistical data, due to their advantages, including a larger spatial scale, wider collection range, and more connected time scale [16,18,19]. As one of the most widely used remote sensing data, NTL data mainly reflect the difference between urban and rural development levels by representing the light brightness of urban buildings and infrastructure at night [20,21]. Due to the characteristics of NTL data, NTL data can directly reflect the economy, population distribution, and development levels between urban and rural areas [22,23]. Since differences exist in the level of economic development between urban and rural areas, these areas can be judged according to these differences, which is reflected by the brightness of night lights, and then the boundary between urban and rural areas can be delineated, which makes NTL data more capable of expressing the heterogeneity of urban–rural space than traditional statistical data [24].

Due to the difference in time scale and spatial resolution, NTL data can be mainly divided into the following three types: the Defense Meteorological Program Operational Linescan System, Suomi National Polar-orbiting Partnership/Visible Infrared Imaging Radiometer Suite, and Luojia-01 data. The time scale of the Defense Meteorological Program Operational Linescan System data is 1992 to 2013, and the spatial resolution is 1000 m. Due to the limitation of the time scale, DMSP/OLS data are less used in current urban space research [25]. Although the spatial resolution of NPP/VIIRS data is only 500 m, which is higher than that of DMSP/OLS data, a spatial resolution of both 1000 m and 500 m is still relatively large for a single city. Therefore, these two kinds of data are mainly applied in macroscopic large-scale studies such as those of urban agglomeration. As a result, the application of these two data in small-scale research of a single city is relatively inadequate [26,27]. In October 2018, Luojia-01, an experimental satellite launched by Wuhan University, began providing free NTL data with a spatial resolution of 130 m to researchers around the world. Compared to DMSP/OLS and NPP/VIIRS data, the improved spatial resolution of Luojia-01 data enables the more detailed study of urban. Conversely, the higher spatial resolution of Luojia-01 also provides a more complete urban spatial structure. Therefore, the current applications of Luojia-01 data have mainly focused on the identification of the urban spatial structure, extraction of urban built-up areas, and delineation of urban–rural boundaries [28,29,30]. However, although the higher spatial resolution of Luojia-01 data has considerably improved the accuracy of urban space research, especially in the delineation of urban–rural boundaries, it is still necessary to further improve the delineation accuracy of urban–rural boundaries due to the disadvantages produced by light spillover in the NTL data [31].

POI data represent urban spatial geographic entities fed back to virtual space. As one of the urban spatial data, POI data can reflect the morphological structure of urban functions by presenting its density agglomeration situation in a geographical space, which has contributed to its increasingly extensive application in urban space [32]. In other words, due to the different development levels of urban and rural areas, significant differences exist in the infrastructure conditions of urban and rural areas. Urban and rural areas can be identified according to the infrastructure conditions, which are reflected by the POI agglomeration density, and then the boundary between urban and rural areas can be delineated. However, POI data have two shortcomings: Although POI data reflect the functional form of a certain area by expressing the size and attributes of density, the development differences of different regions in urban space lead to different density sizes. This is mainly reflected in an obvious trend: a decreasing number of POI agglomerations from the urban area to the rural fringe, which is also why POI is mainly applied in the identification of urban centers [33], delineation of urban functional areas [34], extraction of urban built-up areas [35], and the delineation of urban–rural boundaries. Another shortcoming of POI data is that they are obtained from outside of the actual geographic space of the urban city when calculating the density, which results in the urban form and urban–rural boundaries identified using the POI data being insufficiently realistic. However, with the in-depth study of POI data, it was found that a strong spatial correlation exists between NTL and POI data in urban spaces [36]: the denser the urban geographical entities, the stronger the city light [37]. It was shown in related studies that the data produced by the fusion of NTL and POI data can also be used in related studies of urban space, and the accuracy of the fused data in identifying urban morphology and delineating urban–rural boundaries is considerably higher, which indicates that data fusion will become one of the important directions in future urban studies [38,39].

The main principle of data fusion is to fuse the observation data obtained by different methods for the same target, so that the fused data simultaneously reflect the information characteristics of different data, which produces a more comprehensive and accurate judgment of the study object. As data fusion can improve the use efficiency of data, it has been widely used in urban studies as a method to improve the accuracy of the results [40]. Although the main methods of fusion of NTL and POI data are arithmetic average and weighted fusion, the effects produced by these fusion methods do not reflect the characteristics of the two kinds of data to the maximum extent [41,42,43]. One of the most basic and important data fusion methods is image fusion because image pixel fusion can be analyzed from the perspective of the most basic pixel scale to achieve the best fusion effect. The advantages of wavelet transform, which is used to fuse image pixels on multiple scales and frequencies, make it one of the most important methods for image pixel fusion, being widely used and applied [44,45].

As one of the most important provincial capitals in Western China, as well as one of the cities with the fastest urbanization rate in China in the past decade, Kunming had a high urban and rural development rate of close to 70% at the end of 2019 [29]. However, with the rapid development of the city, a series of urban environmental problems have arisen, for example, the ghost town of Chenggong demonstrates the serious waste of urban resources and the bottleneck in urban construction. Therefore, the accurate delineation of the urban–rural boundary of Kunming will help to better understand the current situation of its urban development, so as to formulate more reasonable planning and construction schemes to promote the intensive use of urban and rural resources and, thus, to achieve the healthy development of Kunming. In this study, NTL and POI data were separately used to delineate the urban–rural boundary of Kunming, and then a new method was constructed. With this method, wavelet transform fusion is used to fuse these two kinds of data for the delineation of the urban–rural boundary. Finally, the results of the urban–rural boundary delineation were further compared and verified in this study.

Kunming is the city with the highest level of urbanization and the most concentrated population in Southwest China. With the proposal and development of China’s One Belt and One Road initiative, the urban construction in Kunming, as the western and central city of China and in Southeast Asia, has accelerated in recent years, and its urban expansion has been obvious. The consequent serious urban and rural environmental problems have seriously challenged the healthy development of the city [40]. Therefore, the accurate delineation of the urban–rural boundary in Kunming in this study will contribute to a better understanding of the current urban and rural areas of the city, which can be used to provide solutions to alleviate the environmental problems generated in the process of urban and rural development and achieve a healthier development of both urban and rural areas in Kunming.

## 2. Materials and Methods

### 2.1. Study Area

**Figure 1:**
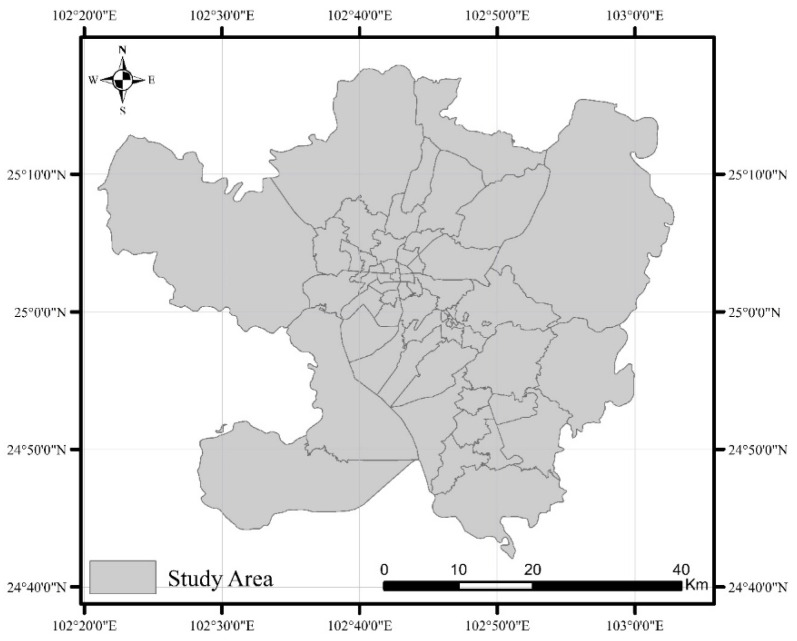
Map of the study area.

Located at 102°10′–103°40′ E and 24°23′–26°22′ N (Figure 1), Kunming has five major urban areas: Wuhua District, Xishan District, Guandu District, Chenggong District, and Panlong District. 

**Figure 2:**
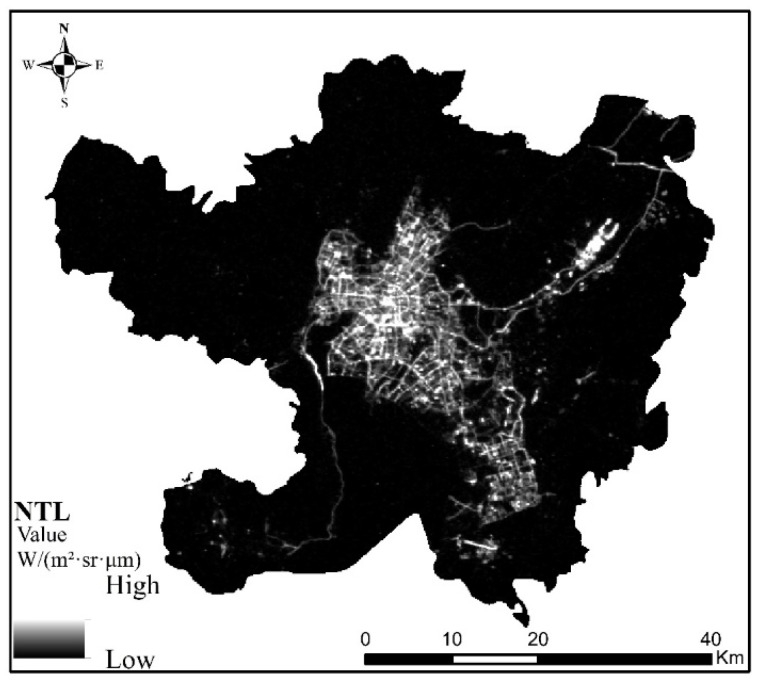
NTL data of Kunming.

The data used in this study mainly included Luojia-01 and POI data, of which Luojia-01 data are available as a free download from the high-resolution earth observation system data of Hubei Province and the application network (http://59.175.109.173:8888/Index.html, accessed on 30 May 2021). The Luojia-01 data of Kunming from October 2018 to October 2020 were obtained by visiting the website on 1 December 2020. Then, after monthly average processing of these data, the NTL data of Kunming were obtained, as shown in Figure 2.

**Figure 3:**
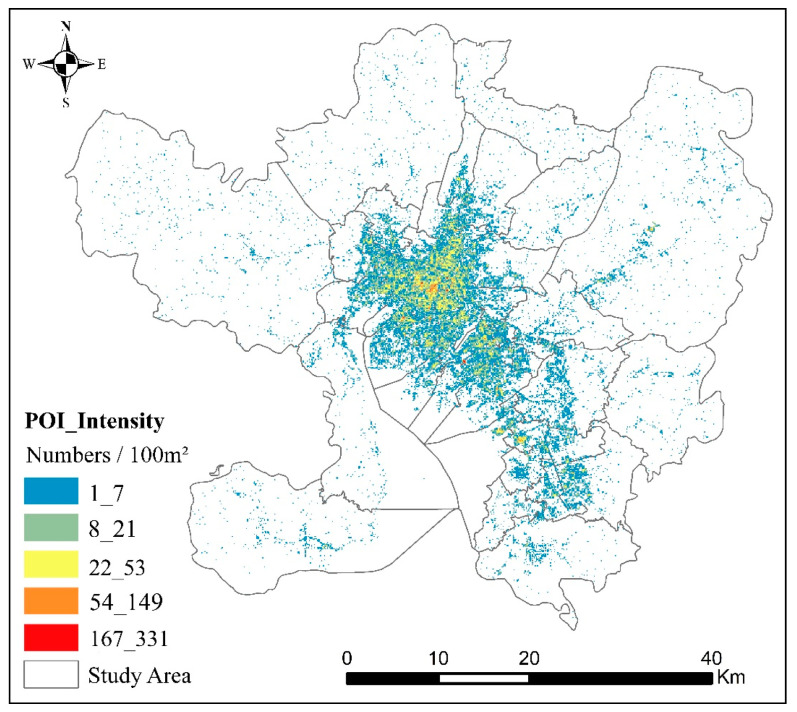
POI data distribution in Kunming.

The POI data can be applied to electronic map navigation, which were obtained from online maps. The electronic map abstracts every building, infrastructure, and road in an urban geographical area into a point-type dataset, including four basic attributes—name, address, coordinate and category—which can be calculated, analyzed, and managed in a geographic information system. Since the aggregation of POI data in the geographic information system represents the amount of infrastructure in the region, it can express the level of infrastructure development in the region, whereas the aggregation of POI with the same attribute can also represent the spatial structure characteristics of different functions of the city. POI data were also obtained by visiting the application programming interface of Amap (https://www.amap.com/, accessed on 2 December 2020). In this study, we obtained 22 and 447,201 categories and quantities, respectively, of POI data in Kunming as of October 2020 through the application programming interface of Amap. Since POI data directly obtained through the interface contained some repeated and meaningless data, such as place names and landmarks, it was necessary to screen, check, filter, and clean the POI data (Figure 3).

### 2.2. Study Methods

#### 2.2.1. Kernel Density Estimate (KDE)

The KDE method mainly reflects the regional differences in physical geographic space by calculating the degree of agglomeration of point and line elements in a virtual geographic space. On the premise of identifying different classification intervals, KDE can combine the similarity value with the maximum inter-category difference [46,47], which enables the urban POI density distribution calculated by KDE to reflect the difference in urban activities between the urban center and the suburbs [48].

(1) $$f\left(s\right)=\overset{n}{\underset{i=1}{\sum }}\frac{1}{j^{2}}k\left(\frac{S-C_{i}}{h}\right)$$

where f(s)  is the function value calculated by the KDE of the spatial position, *j* is the threshold of distance attenuation, *n* is the position in space where the distance is less than or equal to the attenuation threshold, and *k* is the spatial weighting function, in which different search radii *h* have an important impact on the results. Therefore, it was necessary to select an appropriate method to determine the search radius value in this study.

(2) $$h=0.9\times min\left(SD,\;D_{m}\times \sqrt{\frac{1}{ln2}}\right)\times n^{-0.2},$$

where h is the search radius, n is the number of elements in the space, SD is the standard distance, and $D_{m}$ is the median distance.

#### 2.2.2. Wavelet Transform

Wavelet transform is an algorithm that fuses the time domain and the frequency of the image function in image fusion. Compared to other fusion algorithms, wavelet transform can fully consider the interactive relationship between the time domain and frequency of an image by constantly changing the scale under the premise of ensuring the operation in the time domain, which increases the accuracy of the result of image fusion [49]. Additionally, wavelet transform can decompose the image information with the assistance of high-pass and low-pass filters with its unique microscope-type focusing function to unify the steps of the image in the time and frequency domains [50].

The dynamic time-frequency change window of wavelet transform can amplify some part of the image features by localizing the spatial frequency, which enables the image signal to be decomposed into two independent spatial and temporal signals without losing the information contained in the original image signal [51]. The formula of wavelet transform is as follows:

(3) $$WT\left(\alpha ,\tau \right)=f\left(t\right)\varphi \left(t\right)=\frac{1}{\sqrt{\alpha }}f\left(t\right)\overset{+\infty }{\underset{-\infty }{\int }}\varphi \left(\frac{t-b}{\alpha }\right)dt$$

where f(t) is the signal vector, φ(t) is the basic wavelet function, α is scale, τ is translation, and b is a parameter.

**Figure 4:**
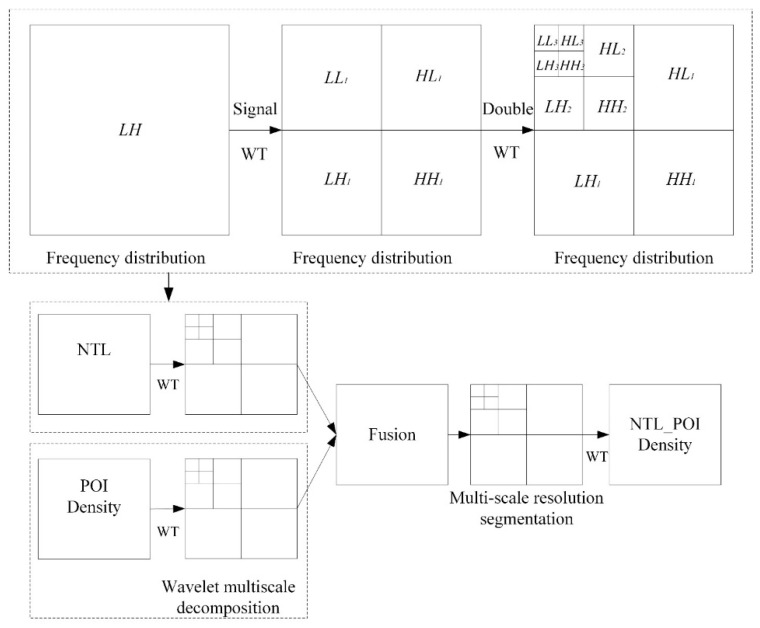
The principle of wavelet transform.

Regarding the principle of wavelet transform, the original image can be regarded as a two-dimensional matrix in the wavelet transform, assuming that the area of the matrix is *N* × *N*, and there is $N=2^{n}$ (where *N* is a non-negative integer). The two-dimensional matrix is broken down into four subregions after each wavelet transform, although the area of the four sub-regions is only 1/4 of the original matrix, as shown in Figure 4. The subregions each contain the wavelet coefficients of the corresponding frequency bands. Therefore, wavelet transform can also be understood as interval sampling in the horizontal and vertical directions [52]. In the second round of wavelet transform, the wavelet coefficients generated by the convolution of the *LL* band in the horizontal direction with a low-pass filter are an approximate representation of the image:

(4) $$f_{2^{j}}^{0}\left(m,n\right)=\langle f_{2^{j-1}}\left(x,y\right),\varphi \left(x-2m,y-2n\right)\rangle$$

The *HL* frequency band is the wavelet coefficient generated by convolution in the horizontal direction with a low-pass filter and then convolution in the vertical direction with a high-pass filter, which represents the singular characteristics of the horizontal direction of the image:

(5) $$f_{2^{j}}^{1}\left(m,n\right)=\langle f_{2^{j-1}}\left(x,y\right),\psi^{1}\left(x-2m,y-2n\right)\rangle .$$

The *LH* frequency band is the wavelet coefficient generated by convolution in the horizontal direction with a high-pass filter and then convolution in the vertical direction with a low-pass filter, which represents the singular characteristics of the vertical direction of the image:

(6) $$f_{2^{j}}^{2}\left(m,n\right)=\langle f_{2^{j-1}}\left(x,y\right),\psi^{2}\left(x-2m,y-2n\right)\rangle .$$

The *HH* band is the wavelet coefficient generated by the convolution of the high-pass filter in the horizontal and vertical directions, which represents the diagonal edge characteristics of the image:

(7) $$f_{2^{j}}^{2}\left(m,n\right)=\langle f_{2^{j-1}}\left(x,y\right),\psi^{3}\left(x-2m,y-2n\right)\rangle .$$

After wavelet transform decomposition, the high- and low-frequency components of the image in different directions could be obtained; the high-frequency component contained the detailed part of the original image, which is locally amplified [53]. Detailed information of the different images was compared in the appropriate wavelet transform domain, fusion was realized in the wavelet domain scale, and finally inverse transformation was conducted (Figure 4).

#### 2.2.3. Multiresolution Segmentation

As an object-oriented bottom-up image segmentation algorithm, multiresolution segmentation is implemented on the basis of the use of an image analysis software such as eCognition. Multiresolution segmentation is mainly used to segment a target image by merging adjacent elements, which can preserve the heterogeneity of the image in the maximum average segment and the homogeneity in the maximum segment, so multiresolution segmentation has been widely used in image segmentation [54].

The scale, shape, and compactness of the target image are determined by multiresolution segmentation with appropriate scale parameters. After the image is multiresolution-segmented using the appropriate scale parameters, the image segmentation effect is the most accurate only when the number of images obtained, the average value of image pixels, and the weighted mean variance of the image area are all the largest. However, the current best method of determining the appropriate scale parameters is visual interpretation, which is inefficient and cannot meet the needs of large-scale image computing. Therefore, researchers have developed a tool named estimation of scale parameters to quickly determine the appropriate scale parameters for image segmentation. The scale parameters determined through this tool improve the efficiency and accuracy of image interpretation [55].

**Figure 5:**
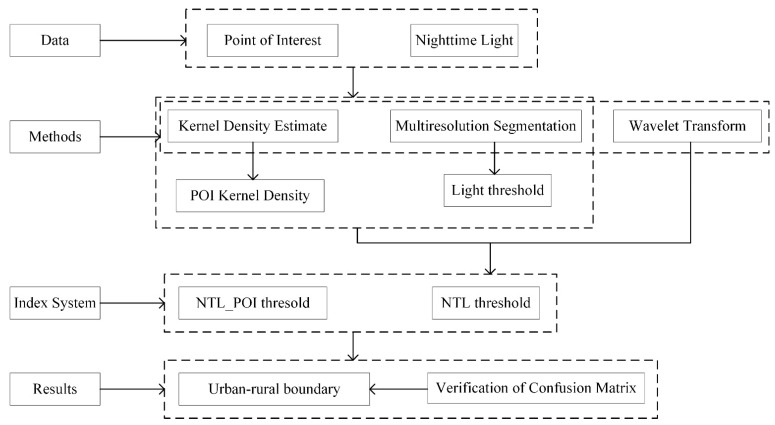
Method for delineation of the urban boundary.

The theoretical framework of research is shown in Figure 5.

## 3. Results

### 3.1. Urban–Rural Boundary Delineated by Different Data

#### 3.1.1. Urban–Rural Boundary Delineated by Luojia-01 Data

The results of Luojia-01 NTL data processing are shown in Figure 2. NTL data of Kunming. From the NTL figure of Kunming, the range of high NTL values is mainly located in Wuhua District, Dongfeng Square of Panlong District, Luosiwan International Trade City of Guandu District, Chenggong University Town, and Changshui Airport of Guandu District. The NTL values of other regions, especially the western part of Xishan District, are relatively low. The distribution of NTL’s range of high and low values also reflects the obvious differences in the level of urban and rural development in Kunming. The scale, shape, and compactness parameters of the multiresolution segmentation determined by estimation of scale parameters are 17, 0.5, and 0.6, respectively.

**Figure 6:**
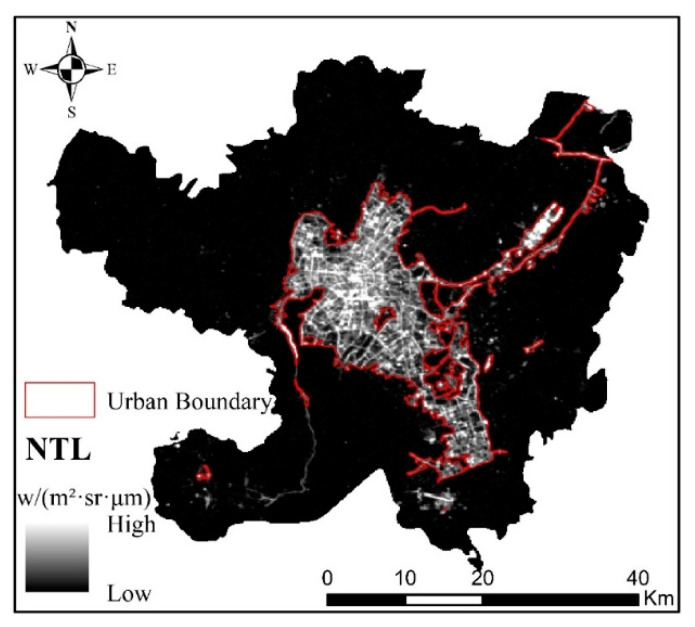
Urban–rural boundary delineated by NTL data.

The NTL image was segmented by the determined segmentation parameters, and the urban–rural boundary was delineated, as shown in Figure 6. We found that the urban areas identified by the NTL data are mainly concentrated in Wuhua District, Dongfeng Square of Panlong District, Chenggong University Town, and Changshui Airport of Guandu District, for a total area of 454.27 km^2^, accounting for 17.37% of the total administrative area. The urban–rural boundaries delineated by the NTL data are severely fragmented and complicated due to the obvious differences in the night-time light in the different areas of the urban space. Therefore, on the whole, the effect of urban–rural boundaries delineated by NTL data is poor.

#### 3.1.2. Urban–Rural Boundary Delineated after the Fusion of POI and Luojia-01 Data

**Figure 7:**
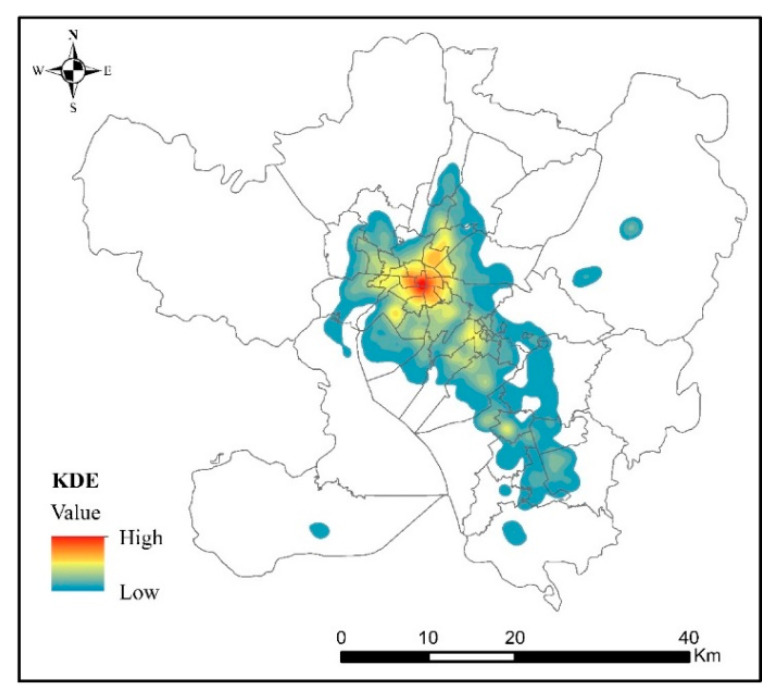
The KDE of POI.

POI data represent the actual unit of each city in a virtual geographic space, so POI data can reflect the development status of urban infrastructure by describing its density agglomeration. Significant differences exist in the density distribution of POI data, especially from the urban center to the fringe and finally to the countryside [56]. Based on the kernel density estimate of POI in Kunming using Formulas (1) and (2), the spatial distribution of POI in Kunming was obtained, as shown in Figure 7. From Figure 6, the POI density distribution shows an obvious circular declining pattern from the urban center to the urban fringe. The POI density is the highest in the urban center, decreasing with increasing distance from the urban center, until it reaches zero. Therefore, we concluded that an obvious correlation exists between the distribution of POI in urban space and the level of urban and rural development, that is, from the urban center to the urban fringe. With a decreasing level of urban and rural development, the POI density also tends to decrease.

**Figure 8:**
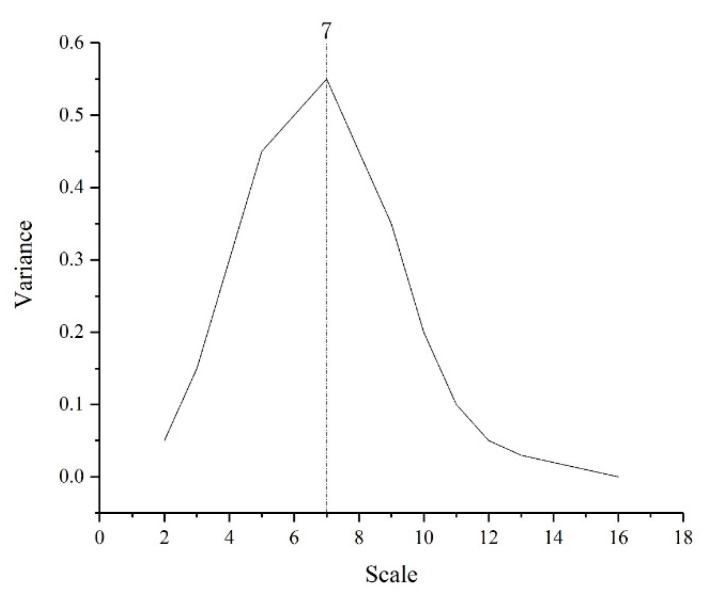
Variation in wavelet coefficient of variance.

POI data reflect the development differences in infrastructure in different areas of the city by describing the aggregation of data in a virtual geographic space, whereas NTL data reflect the level of urban and rural development by describing the brightness of lights in different areas of the city, so a significant spatial correlation exists between the POI density and the NTL brightness in an urban space [57]. This is why we attempted to use wavelet transform to fuse POI and NTL data. The main principle of image fusion is that the fused image can highlight the characteristic parts of the original image. The characteristic part of the original image corresponds to the absolute value of the wavelet coefficient in the process of wavelet transform. In other words, as long as the absolute value of the wavelet coefficient is the maximum, the characteristic part of the original image after wavelet transform is the most obvious, that is, the image fusion can achieve the best effect. When POI and NTL data were fused, the optimal scale of image fusion in this study determined by the difference in wavelet transform coefficients generated by different data was seven (Figure 8).

**Figure 9:**
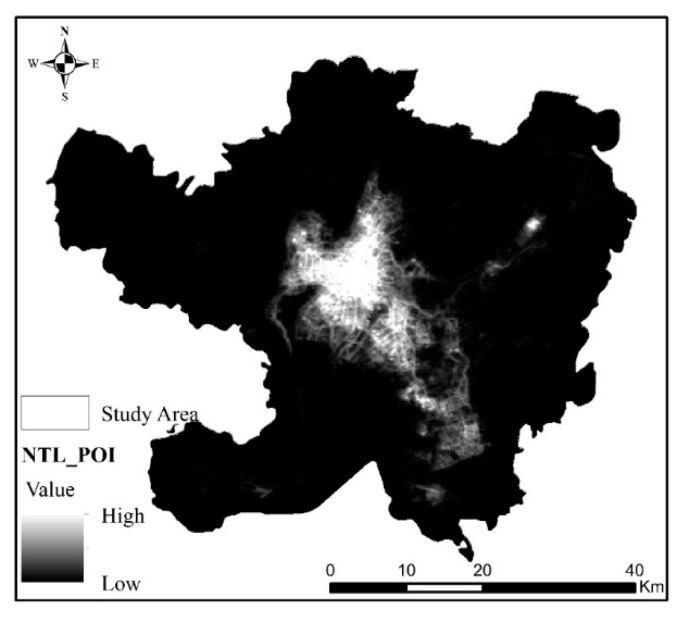
Fusion of NTL and POI data.

**Figure 10:**
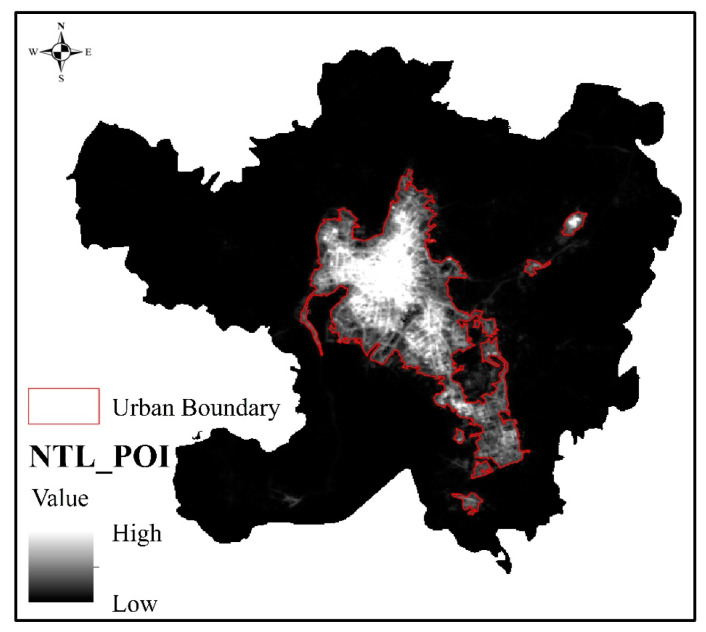
Urban–rural boundaries delineated by NTL_POI data.

The image fused by wavelet transform is shown in Figure 9. After using multiresolution segmentation to segment the fused NTL_POI image, we found that the scale parameters scale, shape, and compactness determined by the estimation of scale parameters were 16, 0.5, and 0.4, respectively. According to the determined scale parameters, the urban–rural boundary could be delineated, as shown in Figure 9. Figure 10 shows that the urban area identified by NTL_POI data mainly concentrated in Dongfeng Square of Panlong District, Luosiwan International Trade City of Guandu District, Chenggong University Town, and Changshui Airport of Guandu District, with a total area of 427.93 km^2^, accounting for 16.36% of the total administrative area. From the results of the urban–rural boundaries delineated by NTL_POI data, the NTL_POI fusion data comprehensively considered the differences in urban and rural development levels and urban development within the region, which resulted in the identified urban scope being more complete and boundary information being more abundant due to the correction of the fragmentation of the urban–rural boundaries.

### 3.2. Comparative Analysis of Data

**Figure 11:**
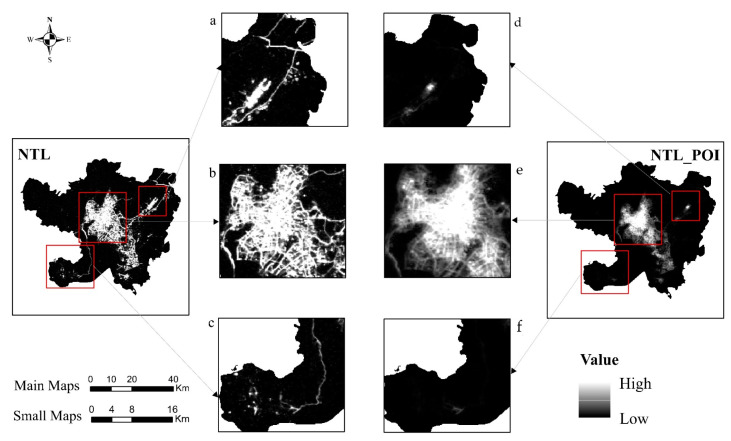
Comparison before and after data fusion ((**a**,**d**) are the airport areas identified by NTL and NTL_POI data respectively; (**b**,**e**) are Dongfeng Square and Guandu Ancient Town identified by NTL and NTL_POI data respectively; and (**c**,**f**) are Xishan Villa Area identified by NTL and NTL_POI data respectively).

Figure 11 compares the results from before and after data fusion. By analyzing Figure 10, it can be found that many similarities exist between the urban boundaries delineated by NTL and NTL_POI data. Firstly, the spatial structures of Kunming reflected by these two kinds of data are similar; secondly, the distribution ranges of high and low values of NTL and NTL_POI data from the urban center to the fringe to the countryside are also similar, that is, the range of high values is mainly distributed in the vicinity of Dongfeng Square, Luosiwan, University Town and Changshui Airport, while in other regions the distribution is mainly medium and low, which indicates that the NTL_POI fused data fully retained the characteristics of NTL data and can reflect the urban and rural development level within the city by describing the brightness value of light.

By comparing NTL_POI data to NTL data, we found that in the actual process of delineating urban–rural boundaries, NTL data only determined whether the region belongs to urban or rural areas by describing the threshold of light brightness, which causes large errors to a certain extent. For example, light brightness is high for main roads, airports, ports, and other areas of the city, which produce an obvious brightness contrast with the surrounding urban areas in the delineation of urban–rural boundaries, resulting in errors in the delineation of urban–rural boundaries. In the vicinity of Changshui Airport, although the light intensity is high and the light range is wide, only a small part of the area was classified as an urban area, and most of the area is an uninhabited area such as the airport runway and ancillary facilities, as shown in Figure 11a. In addition, the light values near the main traffic network are significantly contrasted with the surrounding areas, which resulted in the traffic road network being delineated into an urban area, especially the main road leading to Xishan District, as shown in Figure 11c. However, although most residential areas, shopping malls, and other areas are located in the urban center, only a few lights are produced at night, which created obvious light holes in the NTL image. These light holes fragment the internal space of the urban city, thereby affecting the actual scope of the city and the delineation of the urban–rural boundary. This is also a concentrated manifestation of the difference in the level of urban and rural development in the process of urbanization.

NTL_POI data, which fuses POI data, maintained the characteristics of NTL data while adding the development differences in urban infrastructure reflected by POI data. Therefore, NTL_POI data not only fully considered the level of the light value in an area, but also compared the concentration of urban POI density when delineating the urban–rural boundary, which increased the clarity of the urban scope and urban–rural area in the main urban traffic network, airport, and internal space. Therefore, we concluded that although NTL data can represent the spatial structure within a city by reflecting the level of urban and rural development, due to the limitations of NTL data the data can also produce high values in non-urban areas, which has a stronger impact on the scope of the city. However, NTL_POI data fused with POI data considered urban functions based on urban light brightness, so that NTL_POI data could comprehensively consider the aggregation degree of POI in areas with significant differences in light values, which plays a key role in complementing the detailed information of urban interior space and fringes.

### 3.3. Comparative Verification of Urban–Rural Boundary Delineation Results

**Figure 12:**
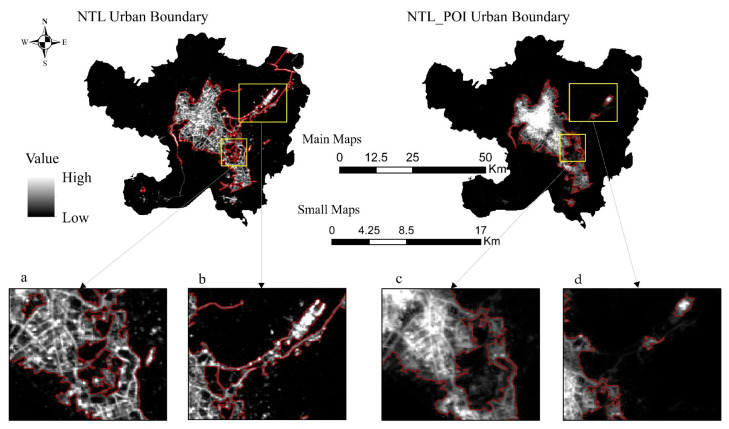
Comparison of urban–rural boundary delineation results ((**a**,**c**) are the airport part of the urban-rural boundary delineated by NTL and NTL_POI respectively; (**b**,**d**) are the Guandu ancient town and Luosiwan part of the urban-rural boundary delineated by NTL and NTL_POI respectively).

By comparing the urban–rural boundaries delineated by NTL and NTL_POI data in Figure 12, it can be found that, firstly, the urban areas delineated by NTL and NTL_POI data are 454.27 and 427.93 km^2^, respectively, accounting for 17.37% and 16.36% of the total area of the administrative region, respectively. Secondly, from the perspective of urban–rural boundaries, although the urban–rural boundary delineated by NTL and NTL_POI data are all mainly concentrated in the vicinity of Changshui Airport, Luosiwan, and Guandu ancient town, the urban boundary delineated by NTL data is larger than that delineated by NTL_POI data. Thirdly, in the vicinity of Changshui Airport (Figure 12a,d), the urban boundary delineated by NTL is far larger than that delineated by NTL_POI data because although functional urban facilities near Changshui Airport are lacking, there are strong lights, so most airport runways are classified as urban area by NTL data. Additionally, in Guandu ancient town (Figure 12b,c), an urban void phenomenon occurs in the urban–rural boundary delineated by NTL, so NTL data did not delineate Guandu ancient town as an urban area due to its low light value at night. However, Guandu ancient town is the main urban area in Kunming. Notably, significant differences also exist between the urban and rural boundaries delineated by NTL and NTL_POI data, resulting from the fact that Luosiwan, as an area connecting the main urban area of Kunming and Chenggong New District, is still under construction. Therefore, although the light value here is relatively strong at night, the development of urban infrastructure is slow.

Therefore, we concluded that since the NTL data only consider the light intensity of a region when delineating the urban–rural boundary, in airports, ports, major traffic networks, and construction areas with strong lights, the delineated urban–rural boundaries will be larger than the actual urban–rural boundaries, whereas in residential areas and shopping malls with weak lights, the boundaries will be smaller than the actual urban–rural boundaries. The data fused with POI data not only comprehensively consider the development difference between light intensity and urban infrastructure, but also consider the influence of the development state of urban infrastructure on the urban scope and urban–rural boundary in the region with an obvious light intensity contrast, which increases the accuracy and reliability of the urban–rural boundary division.

In this study, 1000 random pixel points located in Kunming were selected to verify the urban–rural boundaries delineated by NTL and NTL_POI data. We found after on-site confirmation with Google Earth that among the 1000 selected pixel points, 181 pixel points were located in urban areas and 819 pixel points were located in rural areas. The verification results of the urban–rural boundary confusion matrix delineated by NTL and NTL_POI data after the verification of random pixel points are shown in Table 1. From Table 1, the accuracies of the urban–rural boundary delineated by NTL and NTL_POI data are 84.20% and 93.20%, respectively, and the Kappa values are 0.6549 and 0.8132, respectively. From the perspective of the overall accuracy of urban–rural boundary delineation, after the fusion with POI data the accuracy of urban–rural boundary delineation improved by 9% and the Kappa value improved by 0.1583. Therefore, we concluded that the accuracy of urban–rural boundary delineation was improved significantly compared to the data without fusion, which proves that NTL_POI data have a higher accuracy in urban–rural boundary delineation. Therefore, overall, NTL_POI fused with POI data are more authentic and reliable than urban–rural boundaries delineated by NTL data alone, and the delineation accuracy is also significantly improved.

## 4. Discussion

The rapid and disordered expansion of cities has led to a series of urban problems, which have seriously challenged urban planners when making urban development decisions. As a result, the intensive use of urban resources and the rapid and healthy development of cities have become priorities that cannot be ignored in urban planning. Delineating the boundary between urban and rural areas is an important step in urban planning, which is helpful in limiting the urban sprawl from the aspect of policy making so as to achieve the healthy development of an urban city. On the basis of describing the characteristics of NTL and POI data in an urban space, as well as combining the advantages and disadvantages of the two types of data in urban and rural environment applications, we constructed a new method of using wavelet transform to fuse NTL and POI data to delineate the urban–rural boundary. In addition, through the comparison and verification of the research results, we proved that the fusion of NTL_POI with POI data by wavelet transform produces a significant improvement in the accuracy of delineating urban–rural boundaries.

As data that can directly reflect the level of urban and rural development, NTL data have been important basic data for the study of urban space, including the delineation of urban–rural boundaries [58]. With the deepening of research on NTL data, new methods have been applied to the study of NTL data. However, due to the problems due to light overflow and the oversaturation of NTL data, new methods provided limited improvement in the accuracy of urban–rural boundary delineation [59]. In addition, studies have shown that the urban functions expressed by POI data can be well-combined with those of NTL data, which play an important role in the extraction of urban built-up areas. Therefore, we used wavelet transform to fuse POI and NTL data at the pixel scale to delineate urban–rural boundaries. Compared to the results before fusion, after data fusion, the accuracy of delineating urban–rural boundaries was 93.20%, which surpasses the accuracy of 90% obtained by studies such as the Global Artificial Impervious Area [60,61]. Accurate fusion of urban and rural development level and urban infrastructure differences can effectively improve the results of urban–rural boundary delineation. The fusion of POI and NTL data by wavelet transform compensates for the shortcomings of NTL data alone in urban–rural boundary delineation, increasing the accuracy of urban–rural boundary delineation.

Traditional urban–rural boundaries often refer to a regional-scale indicator system established using socio-economic data, or urban–rural boundaries that are only delineated by spatial analysis of geographic data. These simple urban–rural boundaries have little guiding significance for the discrimination of urban and rural development levels and the formulation of policies aimed toward healthy urban and rural development. Although the NTL data, reflecting the differences in the level of urban and rural development, are some of the better data for distinguishing urban from rural areas, the results of using a single type of data in the urban space are highly inaccurate. However, POI data can reflect the functional differences between urban and rural areas by describing the amount of POI agglomeration, so the urban–rural boundary can be delineated more accurately after the fusion of the two kinds of data. In addition, we used wavelet transform to fuse POI data and NTL data at the pixel scale. Compared to other data fusion methods, wavelet transform highlights the characteristics of NTL and POI data in urban and rural areas after fusing them, which results in the delineated urban–rural boundary better reflecting the land use contradiction between urban and rural areas. Therefore, the urban–rural boundary delineated by this study has an important guiding value for the accurate identification of urban and rural development levels and the formulation of urban and rural planning policies.

Although we proposed a new method to fuse POI and NTL data, which can significantly improve the accuracy of urban–rural boundary delineation, the research still has certain limitations and needs to be further developed. Notably, the urban–rural boundary will change with ongoing urban and rural development. Therefore, the delineation of the city boundary should be a dynamic process. At present, the accurate delineation of urban–rural boundaries is conducive to the identification of and environmental improvement in urban and rural areas to achieve the healthy development of these areas. However, continuous simulation and prediction of urban–rural boundaries according to the future development of cities must be conducted because only dynamic urban–rural boundaries can generate a greater value for the formulation of urban planning and development policies.

## 5. Conclusions

Accurate delineation of urban–rural boundaries provides an important basis for judging urban and rural areas to limit urban sprawl, improve urban and rural environments, and promote the healthy development of these areas. Based on the use of NTL data to delineate urban–rural boundaries, we proposed an image fusion method based on wavelet transform to fuse NTL and POI data at the pixel scale. By comparing and verifying the confusion matrix, we found that the accuracy of NTL data in delineating urban–rural boundaries is 84.20% and the Kappa value is 0.6549, whereas the accuracy of NTL_POI data in delineating urban–rural boundaries is 93.2% and the Kappa value is 0.8132. Therefore, we concluded that the fusion of NTL and POI data by wavelet transform can not only effectively compensate for the error in NTL data in areas with significant light differences, but can also more accurately delineate urban–rural boundaries with the use of the differences in urban and rural development levels and infrastructure development. 

In this study, urban and rural boundaries were delineated by fusing urban and rural development levels with urban and rural infrastructure; knowing these boundaries helps to restrict urban sprawl, alleviate urban issues, and intensively use urban resources to achieve the healthy development of the city. Moreover, it can assist in the formulation of planning policies to improve the urban and rural regional environment, which can play an important role in guiding urban and rural planning. 

## Figures and Tables

**Table 1:** Verification of confusion matrix for urban–rural boundary delineation.

Data		Urban	Rural	Accuracy	Kappa
NTL	Urban	168	21	84.20%	0.6549
	Rural	145	674		
NTL_POI	Urban	171	10	93.20%	0.8132
	Rural	58	761		

## Data Availability

Data Availability DOI:10.5281/zenodo.4923685.
